# Characterizing Carbon Emissions and the Associations with Socio-Economic Development in Chinese Cities

**DOI:** 10.3390/ijerph192113786

**Published:** 2022-10-23

**Authors:** Zijie Shen, Liguo Xin

**Affiliations:** 1 School of Economics & Management, Fuzhou University, No. 2 Wulongjiangbei Avenue, Minhou Country, Fuzhou 350116, China; 2 School of Management, Shandong University, 27 Shanda Nanlu, Jinan 250100, China

**Keywords:** carbon emissions, city, socio-economic features

## Abstract

Reducing carbon emissions in cities is crucial for addressing climate change, while the city-level emissions of different compositions and their relationships with socio-economic features remain largely unknown in China. Here, we explored the city-level emission pattern from the industrial, transportation, and household sectors and the emission intensity, as well as their associations with socio-economic features in China, using the up-to-date (2020) CO_2_ emissions based on 0.1° grid (10 × 10 km) emission data. The results show that: (1) CO_2_ emissions from the industrial sector were considerably dominant (78%), followed by indirect (10%), transportation (8%), and household (2%) emissions on the national scale; (2) combining total emissions with emission intensity, high emission–high intensity cities, which are the most noteworthy regions, were concentrated in the North, while low emission–low intensity types mainly occurred in the South-West; (3) cities with a higher GDP tend to emit more CO_2_, while higher-income cities tend to emit less CO_2_, especially from the household sector. Cities with a developed economy, as indicated by GDP and income, would have low emissions per GDP, representing a high emission efficiency. Reducing the proportion of the secondary sector of the economy could significantly decrease CO_2_ emissions, especially for industrial cities. Therefore, the carbon reduction policy in China should focus on the industrial cities in the North with high emission–high intensity performance. Increasing the income and proportion of the tertiary industry and encouraging compact cities can effectively reduce the total emissions during the economic development and urbanization process.

## 1. Introduction

Global warming caused by excessive emissions of greenhouse gases such as carbon dioxide has become one of the most important challenges facing mankind in the 21st century [1,2,3]. Rising global temperatures cause sea levels to rise, intensify the frequency of extreme climate events such as floods, droughts, and storms, and adversely affect human health [4,5]. To address climate change and reach the “Paris Agreement” target of limiting the global temperature increase to 1.5 °C, reducing carbon emissions has become the consensus of international communities [6]. Cities are a key contributor to climate change, as urban activities are responsible for 75% of global CO_2_ emissions [7]. Given the necessity of the socio-economic development of cities, achieving low-carbon development in cities is of great significance to global climate change mitigation and improving human well-being.

As China is currently the world’s leading CO_2_ emitter, accounting for approximately 30% of global emissions, China plays an important role in global climate change mitigation and emission reduction [8]. China has set a series of reduction targets, such as reducing emission intensity by 60–65% compared with 2005. However, since China is in the stage of rapid industrialization and urbanization, its carbon dioxide emissions are subject to both domestic and international pressures [9,10]. The coal-dominated energy structure, the low energy use efficiency, and the industrial structure where many cities are dominated by energy-intensive manufacturing have caused great difficulties in achieving the carbon reduction target [11,12]. In particular, while reducing carbon dioxide emissions, China has to solve multiple problems such as poverty alleviation, employment, and lessening regional differences [13,14]. Solutions to these problems rely on economic growth, but there is a certain contradiction between reducing carbon dioxide emissions and developing the economy [15,16]. To deal with such a dilemma, exploring the potential associations between socioeconomic features and carbon emissions is crucial for a win–win strategy toward socio-economic development and carbon reduction. Given the huge regional heterogeneity in terms of economic development, size, and industrial structure, decision makers need city-level information on the characteristics of carbon emissions to design context-specific strategies in China.

Recently, there is a growing body of studies on the spatial characteristics of carbon emissions in China, but due to data limitations, most of them were explored at the province level or for individual cities [9,15,16,17]. In terms of spatial data on carbon emissions, the accounting of carbon emissions in foreign studies is primarily based on gridded data, and the accuracy is generally higher than that of domestic ones [18,19]. For instance, Oda et al. used global point source (enterprise) data and luminous data to establish a global 1 km × 1 km CO_2_ emissions gridded map and explored the spatial characteristics of CO_2_ emissions at the global, regional, and city scales [20]. However, the domestic research on the spatial characteristics of carbon emissions is mainly based on statistical data and lacks bottom-up emission data at a high spatial resolution; thus, the city-level spatial pattern of carbon emissions is barely explored in China [21,22]. A recent study revealed the city-level CO_2_ emissions and relations to GDP growth between 2005 and 2015, which mainly focuses on the total emissions and lacks up-to-date analysis [23]. However, investigating the current impacts of socio-economic factors on different compositions of carbon emissions (industry, transportation, and household) and emission efficiency is necessary for adjusting the ongoing carbon policy.

In this study, we used the 0.1° grid (10 × 10 km) CO_2_ emission data in 2020, established by industrial point sources and other data [24,25], combined with the demographic and economic statistics to deeply excavate the city-level emission pattern and the associations with socio-economic features in China. Specifically, the total carbon emissions, different sectors of direct emissions, indirect emissions, per capita carbon emissions, and emission intensity were discussed, and the socio-economic factors include GDP, per capita GDP, industrial structure, and population density. Our results, based on up-to-date data and detailed carbon emissions, can guide the specific formulation of current city-level policies to achieve carbon reduction in an efficient and targeted way.

## 2. Data and Methods

### 2.1. CO_2_ Emission Data

The CO_2_ emission data in 2020 used in this study cover 340 prefecture-level cities in mainland China, excluding Taiwan Province, Hong Kong, and Macau Special Administrative Regions. The city-level emissions were estimated from the gridded emission data at a 10 km resolution (CHRED), which can be accessed via http://www.cityghg.com/a/data/ (accessed on 3 June 2022). The total carbon emission of each grid is the sum of industrial emissions, household emissions, transportation emissions, agricultural emissions, and indirect emissions, excluding carbon emissions caused by changes in forest land and land use types. Notably, industrial carbon emissions were collected from each industrial enterprise (point source) in the grid cell, and the CO_2_ emissions of each enterprise include the emissions from fuel combustion and production processes:

$$E=\sum M_{fuel}\times F_{fuel}+E_{p}$$

where *E* represents the CO_2_ emission, *M_fuel_* is the amount of a certain fuel, *F_fuel_* is the CO_2_ emission factor of this fuel, and *E_p_* represents the CO_2_ emission in the industrial production process. Among them, the carbon emissions in the industrial production process mainly come from the manufacturing process of cement, lime, steel, and glass. During the data collection of industrial point sources, wrong locations of enterprises have been corrected after comparing the administrative attributes with the actual coordinates, which accounted for about 5.7% of the enterprises.

Different from the calculation of industrial carbon emissions, agricultural, household, and transportation carbon emissions were derived from the statistical data of provinces and regions. More detail on this CO_2_ emission dataset can be found in [25]. Therefore, the carbon emission data used in this study are not only highly accurate, basically covering all industrial point sources, but also comprehensive, which makes up for the statistical data and low-resolution grid data used in previous carbon emission studies and further improves the accuracy and reliability of the results.

### 2.2. Emission Types Based on Total Emission and Emission Intensity

We classified all the Chinese cities into four emission types based on total emissions and emission intensity: (1) High–High, (2) High–Low, (3) Low–High, and (4) Low–Low [26]. Specifically, indicators of total emissions include total emissions, household emissions, and industrial emissions; emission intensity was measured by per capita emissions or per GDP emissions. The high/low was classified by the national median value, and a high emission intensity indicates a low emission efficiency. Taking household emissions per capita as an example, the High–High type refers to the cities with high household emissions and high per capita household emissions, which is the worst type in our assumption, the High–Low type refers to the cities that have high household emissions but low per capita emissions, the Low–High type refers to the cities that have low household emissions but high per capita emissions, and the Low–Low type refers to the cities that have both low household emissions and low per capita emissions, which is the best type.

### 2.3. Statistical Models

The IPAT model has been widely used to explore the impact of human activities on environmental changes [27,28,29], where I represents the impact on the environment, and P, A, and T represent demographic, economic, and technological factors, respectively. However, the IPAT model is relatively simple and fails to reflect the complex impacts on the environment. Therefore, Dietz and Rosa proposed the STIRPAT (Stochastic Impacts by Regression on Population, Affluence, and Technology) model in 1994 [27]:

$$I_{i}=aP_{i}^{b}A_{i}^{c}T_{i}^{d}e_{i}$$

The STIRPAT model not only can be used to evaluate the impact of these three factors on the environment but also can be further decomposed into more influencing factors. The decomposed model is widely used in the study of influencing factors of carbon emissions [30,31].

Based on the summary of existing research and the spatial autocorrelation characteristics of carbon emissions in China, we extended the STIRPAT model from the economic perspective and chose GDP, GDP per capita (PCG), industrial structure (IS), and population density (PD) as independent variables. Those socio-economic features were selected because previous studies have proven that the economic level, population size, and industrial structure are related to carbon emissions in China [9,32,33]. GDP per capita is the ratio of the total regional GDP to the local population [23], the industrial structure is the proportion of the secondary sector of the economy [34], and population density is the ratio of the population to the built-up area of the city [35]. After taking the natural logarithm of variables, the statistical model set in this study is:

ln *y* = α_0_
*C* + α_1_ ln(*GDP*) + α_2_ ln(*PCG*) + α_3_ ln(*IS*) + α_4_ ln(*PD*) + ε

where *y* represents the indicators of CO_2_ emissions, *C* represents the constant term, α represents the corresponding regression coefficient, and ε is the random error of the model. The data on socioeconomic factors are collected from the “China Urban Statistical Yearbook” and the “China Regional Economic Statistical Yearbook”.

## 3. Results

### 3.1. CO_2_ Emission Structure

**Figure 1:**
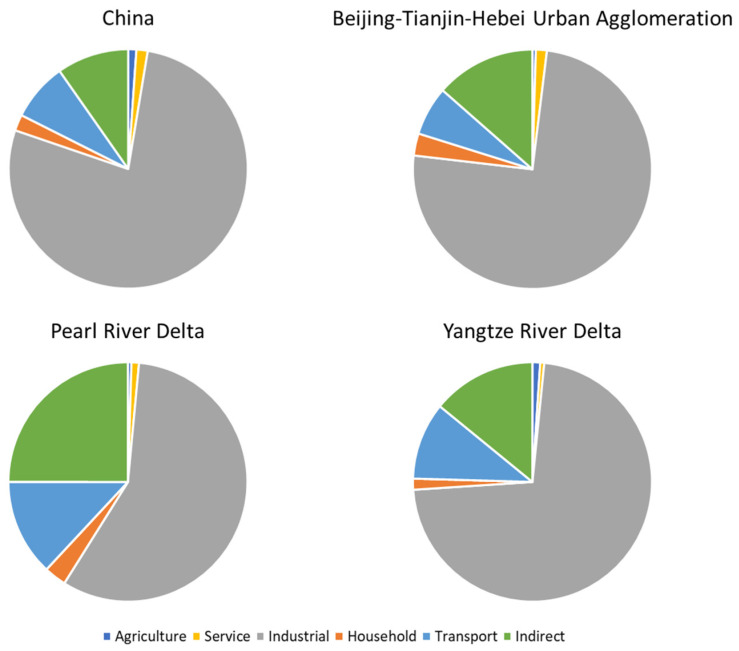
The proportion of CO_2_ emissions in China and the three largest urban agglomerations. Direct emissions consist of emissions from agriculture, services, industry, households, and transportation.

We first analyzed the structure of carbon emissions in China and the three largest urban agglomerations (Figure 1). For the entire country, the CO_2_ emissions from the industrial sector were considerably dominant (78%), followed by indirect (10%), transportation (8%), and household (2%) emissions. Compared to the national emission structure, indirect emissions took a larger proportion in three megaregions, especially in the Pearl River Delta (25%), and the proportion of transportation emissions in the Pearl River Delta (13%) and Yangtze River Delta (10%) was larger, while the proportion of industrial emissions in the Pearl River Delta (57%) was smaller.

### 3.2. Spatial Distribution of Total Emissions and Emission Intensity

**Figure 2:**
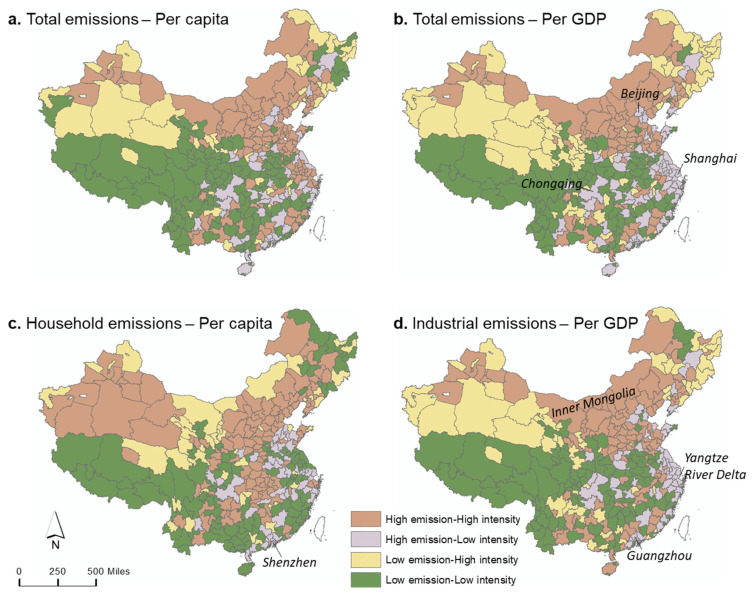
CO_2_ emission types based on total emissions and the corresponding emission intensity. Taking household emissions per capita (**c**) as an example, the ‘high emission–high intensity’ refers to the cities with high household emissions and high per capita household emissions. The high/low was classified by the national median value.

Total CO_2_ emissions and emission intensity are combined in the spatial map (Figure 2a,b), presenting four types of cities. Two total emission–emission intensity maps show similar patterns: high emission–high intensity cities were concentrated in the North, while low emission–low intensity types mainly occurred in the South-West. Cities in the North-West part are primarily low emission–high intensity, which is also notable for the potentially large increase in total emissions in the future. Many developed cities such as Beijing, Chongqing, and Shanghai have high total emissions but low emission intensities. In addition, most coastal cities emitted high total emissions but low emissions per unit of economic output (Figure 2b).

We further presented the emission types in terms of the major sector, industrial emissions (Figure 2d), and household emissions representing residential energy-use behaviors (Figure 2c). For household emissions, most cities with high emissions also have high per capita emissions, indicating a low-efficient behavior of residential energy use. Such noteworthy cities were concentrated in Northern China and Central China. Residential people in megacities such as Shanghai and Shenzhen perform well with low per capita emissions. For industrial emissions, the industrial cities in the North (e.g., Inner Mongolia, Shanxi province, Anshan) primarily belong to the High–High type, but those in the South and coastal cities (e.g., Chongqing, Guangzhou, Yangtze River Delta) were classified as the high–low type with low emission intensities.

### 3.3. Relations between CO_2_ Emissions and Socio-Economic Features

**Figure 3:**
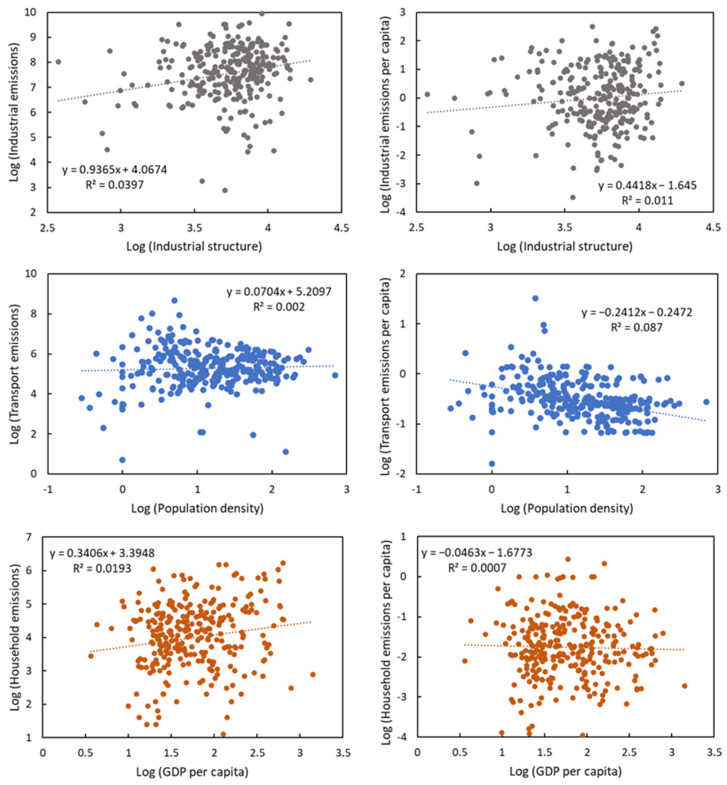
Scatter plots between CO_2_ emissions and socio-economic factors in Chinese cities.

The direct CO_2_ emissions and emission intensities of three major sectors were related to the corresponding socio-economic factors. First, we investigated the relations between industrial emissions and the industrial structure (proportion of the secondary sector of the economy). The industrial structure was positively associated with both industrial emissions and per capita emissions, indicating that a greater proportion of the secondary industry tends to increase the industrial emissions and emission intensity (Figure 3). Second, we explored the relationship between transportation emissions and population density. Cities with a higher population density tend to have more transportation emissions but fewer per capita transportation emissions. Third, higher income levels, represented by GDP per capita, were linked to more household emissions across cities. Overall, economic development and population growth had negative relations to emission intensity.

To further explore the impacts of socio-economic features on CO_2_ emissions, we built multiple regression models on diverse emission sectors and emission efficiency indicators (Table 1). The dependent variables in the regression equation are the logarithm of CO_2_ emissions, and the independent variables are the logarithm of socio-economic features, including GDP, per capita GDP, industrial structure, and population density. Those models were tested significantly, indicating a linear regression relationship between socio-economic features and CO_2_ emissions. According to the fitness reflected by the adjusted R square, socio-economic features can better explain the total emissions and household emissions compared to other emission indicators.

Specifically, cities with a higher GDP and a larger proportion of secondary industry tend to have more total emissions, but those with a higher GDP per capita and population density tend to have fewer emissions. Such effects are also reflected in different sectors of total emissions, i.e., industrial emissions and household emissions, as well as emission efficiencies in terms of population and GDP. Some exceptions exist for the indirect emissions, which consist of the electricity consumption produced in external cities, as they are majorly promoted by GDP. In addition, cities with a higher GDP tend to emit less CO_2_ per capita, and CO_2_ per GDP represents a higher emission efficiency, whereas those with a higher GDP per capita tend to emit more CO_2_ per capita.

Our models also show trade-offs between economic development and CO_2_ emissions, as well as between CO_2_ emissions and emission efficiency. For example, a higher GDP could promote CO_2_ emissions and emission efficiency; higher incomes can reduce emissions but increase per capita emissions. Meanwhile, synergies include more secondary industries associated with higher emissions and a lower emission efficiency, and a higher population density is associated with fewer emissions and a higher emission efficiency.

## 4. Discussion

### 4.1. Complex Relationships between Socio-Economic Development and CO_2_ Emissions

Previous studies have revealed that GDP and industrial structure have a certain role in promoting total CO_2_ emissions [36,37,38]. We investigated more detailed CO_2_ emissions from different sectors and found similar promoting impacts of GDP growth on the industrial, household, and indirect emissions (Table 1). The industrial structure has suppressed industrial carbon emissions to a larger extent than total emissions, indicating that reducing the proportion of secondary industry would play a certain role in alleviating the growth of industrial emissions, which is the major component of total carbon emissions (Figure 1 and Figure 2). For industrialized cities, the improvement of the economic level may lead to an increase in carbon emissions. However, economic development is often accompanied by technological progress and the optimization of the industrial structure [39,40]. This can be implied by the negative correlation between GDP and per GDP emissions that we found. Therefore, developing the economy in a sustainable manner, such as increasing the proportion of tertiary industry and improving technological progress, could contribute to carbon reduction effectively.

Economic development in a city is reflected not only in the GDP growth but also in the rising income level [41]. We found positive impacts of GDP on carbon emissions, which is consistent with previous research [16]. In contrast, our models detected negative impacts of the income level, represented by GDP per capita, on CO_2_ emissions, especially on household emissions (Table 1), implying that a higher income would lead to fewer CO_2_ emissions. Although the EKC curve presents a non-linear relationship between environmental degradation and GDP per capita [42,43], our models show positive correlations between per capita emissions and GDP per capita, which is consistent with the previous long-term research in Chinese cities [23]. This might imply that developed Chinese cities are about to reach the peak of GDP per capita, which is the inflection point towards a negative relationship [23]. Such positive relationships may be due to the increase in the income level being related to the improvement of consumption capacity and living standards, which would increase the load on resources and the environment [44,45]. Overall, an improved income level is beneficial for total carbon reduction, whereas the high per capita energy consumption by high-income groups is noteworthy.

Population growth is another trend associated with economic development [46]. We observed that a higher population density was associated with fewer CO_2_ emissions and a lesser emission intensity. Similarly, previous research for 154 countries yielded a significant negative correlation between population density and carbon dioxide emissions. This is a piece of evidence supporting the building of compact cities. In contrast, a previous study found positive impacts of population density on per capita emissions for the 30 provinces in China during the period 1995–2012. This contradiction might be explained by the different spatial and temporal scales. Recently, many urban scientists have claimed that compact cities are sustainable [47,48], and our results also indicate that compact cities represented by a higher population density tend to emit less CO_2_ and have a lower emission intensity. We also observed that fewer transportation emissions per capita are associated with a higher population density (Figure 2), indicating low carbon emission transportation in compact cities [49]. Thus, compact forms of cities might be an effective way to achieve carbon reduction during urbanization in China.

There are several limitations in our statistical models explaining CO_2_ emissions by socio-economic features. The STIRPAT model used in this study only concerns the linear relations, and, thus, future research could consider nonlinear regression and the interaction between independent variables, as well as stepwise regression models or principal component analysis to obtain the major influencing factor. Additionally, the trade structure in which exports are dominated by energy-intensive products and imports are dominated by high-value-added products is another economic factor that influences carbon emissions [11,12]. Despite the economic side, the characteristics of CO_2_ emissions are largely determined by the natural resource endowment and the structure of cooking energy consumption [50,51]. Future research can involve more control variables such as energy structure, heating days in the winter, and cooling days in the summer in order to explore more accurate impacts of economic development on CO_2_ emissions.

### 4.2. Implications for Carbon Reduction Policy

Our study provides essential and detailed information on CO_2_ emissions from specific sectors and spatial distributions in order to inform policymakers of effective carbon reduction actions in China. First, our maps of CO_2_ emissions and emission efficiency detected the most noteworthy cities for carbon reduction, which are those with both high emissions and a high emission intensity (Figure 2). We found that these high emission–high intensity cities are mostly located in regions with geographical advantages and energy advantages such as abundant coal, mines, or oil field resources, but they have a low degree of economic development due to traditional industry. In particular, the cities with high per capita emissions excessively depend on natural resources, and they are important bases for resource production in China.

Second, our models reveal how to reduce CO_2_ emissions from a specific sector and improve emission efficiency. Specifically, decreasing the proportion of secondary industry can significantly lessen industrial emissions, and this can be achieved by developing diverse tertiary sectors of the economy such as tourism, financial, and real estate activities based on the existing geographical advantages of industrial cities [52]. To reduce household emissions, high-income groups are worthy of attention, since income levels can increase household emissions. The extensive utilization of energy can result in a waste of energy and a large increase in carbon emissions [45]. Additionally, enhancing emission efficiency by reducing secondary industry and compacting population distribution is crucial for controlling carbon emissions in long-term urban sustainable development [53]. Thus, developing the economy in a sustainable manner, such as increasing the proportion of tertiary industry and improving technological progress, could contribute to both carbon reduction and economic growth.

## 5. Conclusions

In this study, we analyzed the characteristics of CO_2_ emissions and emission efficiency for 340 Chinese cities and further explored their associations with socio-economic features in order to recognize the most effective way to reduce CO_2_ emissions by targeting specific sectors and cities. We found that industrial emissions are the dominant component of total CO_2_ emissions for the country and large urban agglomerations. The spatial maps indicate that industrial cities in the North with both high emissions and a high intensity are the most noteworthy regions, highlighting the importance of considering the total emissions and efficiency simultaneously when designing carbon reduction policies. The GDP, GDP per capita, industrial structure, and population density were significantly related to CO_2_ emissions and emission intensity. Although a higher GDP was related to more CO_2_ emissions, a higher GDP and a higher income were related to lower per GDP emissions. A higher income is also related to fewer CO_2_ emissions but could increase per capita emissions. Reducing the proportion of the secondary sector of the economy and increasing population density could significantly decrease CO_2_ emissions and emission intensity. These findings suggest that encouraging the tertiary sector of the economy and developing compact cities are effective actions that can both achieve the carbon reduction target and not hinder the economic development and urbanization process in China.

## Figures and Tables

**Table 1:** Linear regression models for CO_2_ emissions and emission intensity.

	Total Emissions	Industrial Emissions	Household Emissions	Indirect Emissions	Per Capita Emissions	Per GDP Emissions
Constant	1.98 ***	0.62	−1.73 **	1.487	1.98 ***	1.97 **
GDP	0.74 ***	0.63 ***	0.97 ***	0.866 ***	−0.26 ***	−0.26 ***
GDP per capita	−0.54 ***	−0.56 **	−1.24 ***	−0.58	0.46 ***	−0.54 ***
Industrial structure	0.46 **	1.01 ***	0.24	−0.28	0.46 **	0.46 **
Population density	−0.36 ***	−0.48 ***	−0.26 **	−0.24	−0.36 ***	−0.36 ***
Adjusted R^2^	0.42	0.27	0.44	0.31	0.22	0.24

Note: *** and ** indicate significance levels of 1% and 5%, respectively.

## Data Availability

The data and materials are available from the corresponding author upon reasonable request.
